# A first-principles investigation of α, β, and γ-MnO_2_ as potential cathode materials in Al-ion batteries

**DOI:** 10.1039/d0ra08401h

**Published:** 2020-11-02

**Authors:** Joshua Fu, Xuan Luo

**Affiliations:** National Graphene Research and Developmental Center Springfield Virginia 22151 USA

## Abstract

An inexpensive and eco-friendly alternative energy storage solution is becoming more in demand as the world moves towards greener technology. We used first principles calculations to investigate α, β, and γ-MnO_2_ and their Al-ion intercalation mechanism in potential applications for aluminum batteries. We explored these complexes through investigating properties such as volume change, binding/diffusion energy, and band gap to gauge each material. α-MnO_2_ had almost no volume change. γ-MnO_2_ had the lowest binding energy and diffusion barrier. Our study gives insight into the feasibility of using MnO_2_ in aluminum batteries and guides investigation of the material within its different phases.

## Abstract 2

An inexpensive and eco-friendly alternative energy storage solution is becoming more in demand as the world moves towards greener technology.

## Graphical abstract

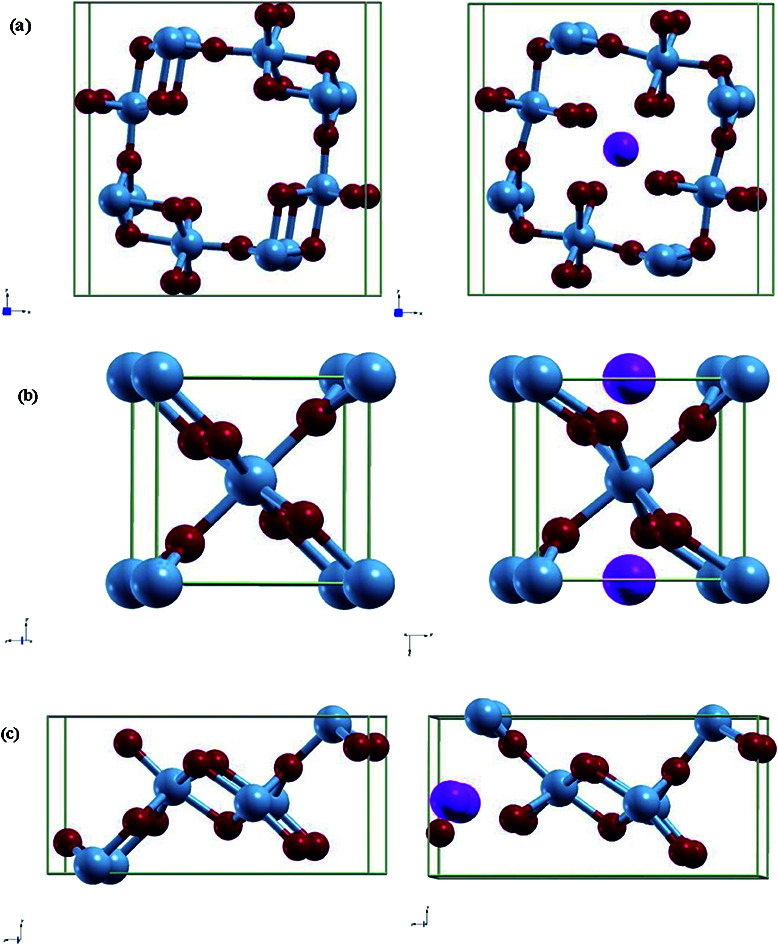

## 1 Introduction

The current rate of energy consumption relying on current energy production methods is unsustainable.^1^ Specifically, the use of fossil fuels will be extremely limited in the long term due to environmental concerns. To combat this problem, renewable energy sources are being harnessed as solutions. The most common examples are wind, solar, geothermal, biomass, and hydropower.^2^ As these sources are intermittent, the importance of efficient energy storage cannot be understated; batteries are a major necessity.^3^ Lithium-ion batteries (LIB) are the current go-to energy storage solution because of their high energies and power densities and voltage to volume ratio stemming from the ions' natural large electrochemical potentials. However, current variations of LIBs are facing issues of high cost, potential safety issues, and low capacity.^4,5^ Several reports have addressed these issues by substituting toxic active materials with non-toxic ones and flammable non-aqueous electrolytes with aqueous ones.^6–8^ Motivated by these substitutions, sodium, magnesium, potassium, calcium, zinc, and aluminum-ion batteries are some of the alternatives that have seen some development with aqueous electrolytes as well.^9–13^

Aluminum-ion batteries (AIB) are currently being investigated for having high volumetric capacity, low cost, and high safety.^14,15^ These properties owe themselves to its three electron redox properties and smaller volume, abundance in the Earth's crust, and low-relative reactivity. Compared to Li^+^, Al^3+^ has a significantly smaller radius and its strong electrostatic interactions/high charge density makes the insertion/extraction with the host structure more difficult.^16^ This obstacle and the potential of Al-ion batteries is high make the search for materials that are capable of inserting these ions essential. Dai *et al.* explored a graphitic foam cathode, showing a high voltage, moderate storage capacity, and high charge/discharge cycle stability.^17^ This experiment created an interest in graphene applications within AIB. Further investigation in novel electrolytes showed improvised cycling performance and discharge capacities through the chloroaluminate anions intercalation mechanism.^18^ Modifications to the cathode/anode material structure for further improvement included using composites, multilayers, bilayers, flakes, microspheres, *etc.*^19,20^ Other popularly-explored materials include various metal oxides, sulfides, and selenides.^16,21^

Manganese dioxide (MnO_2_) has received significant attention owing to its electrochemical properties, low cost, and environmental benefits.^22^ It exists in several allotropes, namely with the prefixes α, β, δ, γ, λ, and others. All of them have the same octahedral arrangement of atoms, but are defined by different connections of the basic structural unit, the MnO_6_ octahedron. Natural tunnels exist within the crystal structure, making them suitable for the ion intercalation mechanism within batteries. In the context of AIB, each polymorph have been investigated experimentally.^23–27^ Theoretically, α-MnO_2_ has been systematically researched.^28,29^ Juran *et al.* calculated and compared properties such as voltage and volume change of commonly used MnO_2_ phases, including α-MnO_2_, β-MnO_2_, δ-MnO_2_, γ-MnO_2_, and λ-MnO_2_. Each phase was investigated with ions commonly used in batteries intercalated within.^30^

Although the electrochemical properties of the various morphisms of MnO_2_ have remained largely unexplored with Al-ions, there are precedents of extensive study into its application as a cathode in many other battery types. In Mg-ion and Zn-ion batteries, favored phases of MnO_2_ have emerged for each type of battery among researchers.^31–33^ Mg-ions generally have had promising adsorption qualities with δ-MnO_2_ and α-MnO_2_.^9,34^ On the same note, α-MnO_2_, β-MnO_2_, and δ-MnO_2_ are promising cathode materials used in Zn-ion batteries.^33,35,36^ It is not yet easily predictable what morphology is most suitable for Al-ion intercalation. In this paper, we will explore α, β, and γ-MnO_2_ as potential cathode materials for Al-ion batteries, chosen for their presence in research for other battery types and their natural tunnels within their structures. We will investigate electrochemical properties of each MnO_2_ morphology and give insight into its compatibility with the intercalation mechanism in Al-ion batteries. We will use first-principles calculations to research unexplored properties such as density of states, binding energy, and the diffusion barrier. As we are analyzing trends in these different properties, we will be using DFT rather than the DFT+U used in closely related papers, which may provide a new perspective.^30^ The second section of this paper, the method, details the process of computation and analysis. The third section, the results and discussion, will give a thorough analysis on the result obtained through the process described in the method. The last section, the conclusion, will highlight key points observed in the results and reiterate it in context of the motivation behind this paper.

## 2 Method

### 2.1 Computational details

We performed first-principle calculations based on Density Functional Theory (DFT) with Perdew–Burke–Ernzerhof (PBE) parameters using the Generalized Gradient Approximation (GGA) functional implemented using ABINIT to depict exchange-correlation energy.^37^ We used the projector-augmented-wave (PAW) method. The PAW pseudopotentials used were generated with valence electron configurations and radius cutoffs.

The unit cell used orthogonal primitive vectors. The planewave-basis set and the Brillouin zone was measured using the converged values obtained by stopping SCF cycles when the change in total energy became 1.0 × 10^−10^ Ha. Convergence was done for undoped polymorphs, and the same converged values were used for doping. Converged values varied between different structures and are listed in Table 1.

**Table 1:** Convergence for energy cutoff and *k*-point mesh

	α-MnO_2_	β-MnO_2_	γ-MnO_2_
Energy cutoff (hartree)	16	20	18
*k*-Points	16 × 8 × 8	16 × 8 × 8	8 × 4 × 4

The lattice parameters of both the unit cell before and after adsorption were relaxed using the Broyden–Fletcher–Goldfarb–Shanno minimization (BFGS) using double relaxation method of first the ionic positions of the atoms and then a dilatation of the three lattice parameters.^38^ Optimizations were done with SCF cycles stopping when consecutive iterations had a force difference of 1 × 10^−5^ Ha/Br and a maximal absolute force tolerance in which BFGS structural relaxation iterations will stop of 1 × 10^−6^ Ha/Br.

### 2.2 Atomic structure

**Fig. 1:**
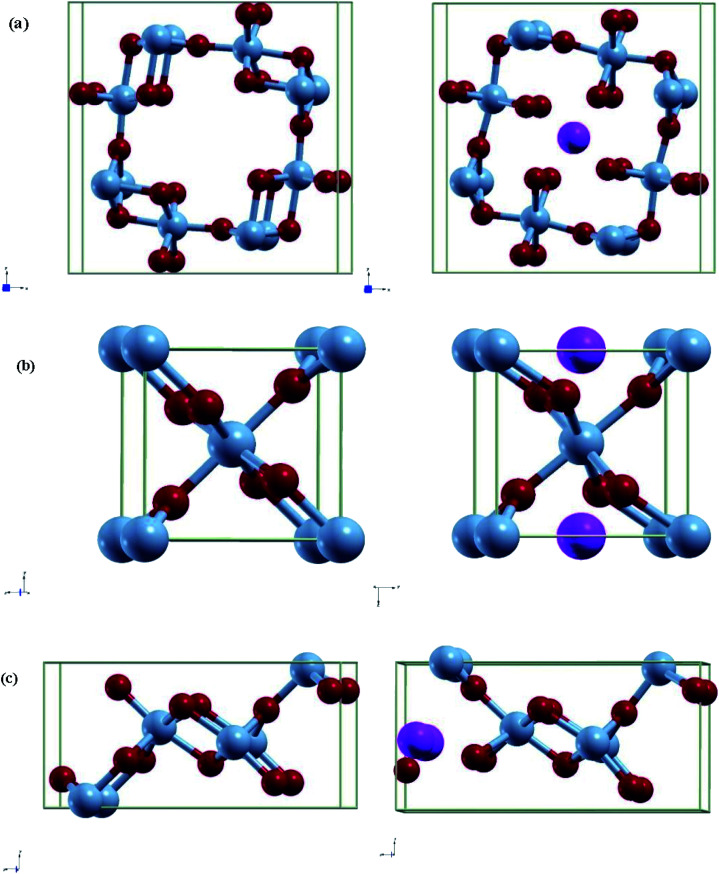
Relaxed structures of pre/post adsorption of Al-ions in (a) α-MnO_2_, (b) β-MnO_2_, and (c) γ-MnO_2_. Blue spheres are Mn, red spheres are O, and pink spheres are Al.

A 1 × 1 unit cell was constructed for the double relaxation described in the previous subsection. The adsorption site for Al was found after comparing total energy of adsorption for each possible site, which were the possible Wyckoff sites for each MnO_2_ phase. The sites found for each phase are shown in Fig. 1. For each relaxed polymorph before and after adsorption, total volume change are calculated and compared. The volume change equation is that as follows:1
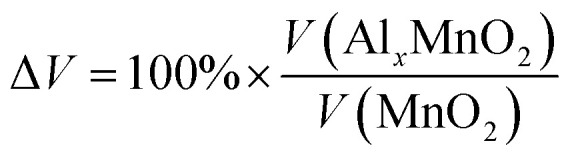
where *V* is the volume of the solid.

### 2.3 Electronic structure

The band structure and the projected density of states (PDOS) were calculated for each phase with and without adsorption. The atoms chosen for projection were the atoms closest to the site of intercalation. From these we can analyze the electronic structure for each polymorph.

### 2.4 Binding/diffusion energy

The binding energy can be found through the equation:2
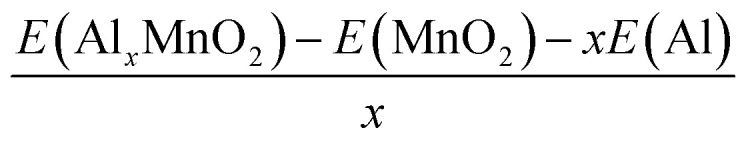
*E*(Al_*x*_-MnO_2_) is the total energy of the polymorph adsorped with Al. *E*(MnO_2_) is the total energy of the pure polymorph, *E*(Al) is the total energy of aluminum metal. This equation is also the equation of choice in other papers as well.^39^ The diffusion energy is calculated by placing the Al-ion at different *c* coordinates and comparing total energy. The structure used for total energy calculation is based on the double relaxed structure after adsorption. The results are then normalized so that the lowest energy is at 0. The *c* coordinates are chosen based on 1/16th increments of the fractional coordinates.

## 3 Results

### 3.1 Structural properties

Each MnO_2_ structure is made up of tunnels and MnO_6_ octahedron. α, β, and γ consists of 2 × 2, 1 × 1, and 2 × 1 tunnels and its conventional cell contains 24, 6, and 12 atoms respectively. These differences in the space of their tunnels influence the extent of volume change during intercalation. Both the relaxed lattice parameters and the volume change are noted in Table 2 and the corresponding relaxed structures are shown in Fig. 1.

**Table 2:** Lattice parameters

	α-MnO_2_	α-Al_0.125_-MnO_2_	β-MnO_2_	β-Al_0.5_-MnO_2_	γ-MnO_2_	γ-Al_0.25_-MnO_2_
*a*/*b* (Å)	9.68	9.48	4.45	4.60	2.87/4.51	2.95/4.64
*c* (Å)	2.86	2.80	2.94	3.04	9.28	9.55
Δ*V* (%)	0	−6.2	0	10.5	0	9.9

Looking at the volume change is crucial in looking for possible effects of long-term strain and eventually cracking of the cathode material. In this regard, we see that the volume change for α is negative, while β and γ are both positive with the volume change in β being more pronounced. As α has a 2 × 2 tunnel, which is much larger compared to γ or β, Al-ions may have a different effect on the volume change of the unit cell due to the increased distance between the ion and other atoms. The trend stated is the same as reported in other papers.^30^ Furthermore, the lattice parameters and bond lengths obtained were very close to some studies that used other methods such as adding a Hubbard correctional to do relaxation.^40,41^

### 3.2 Electronic structure

**Fig. 2:**
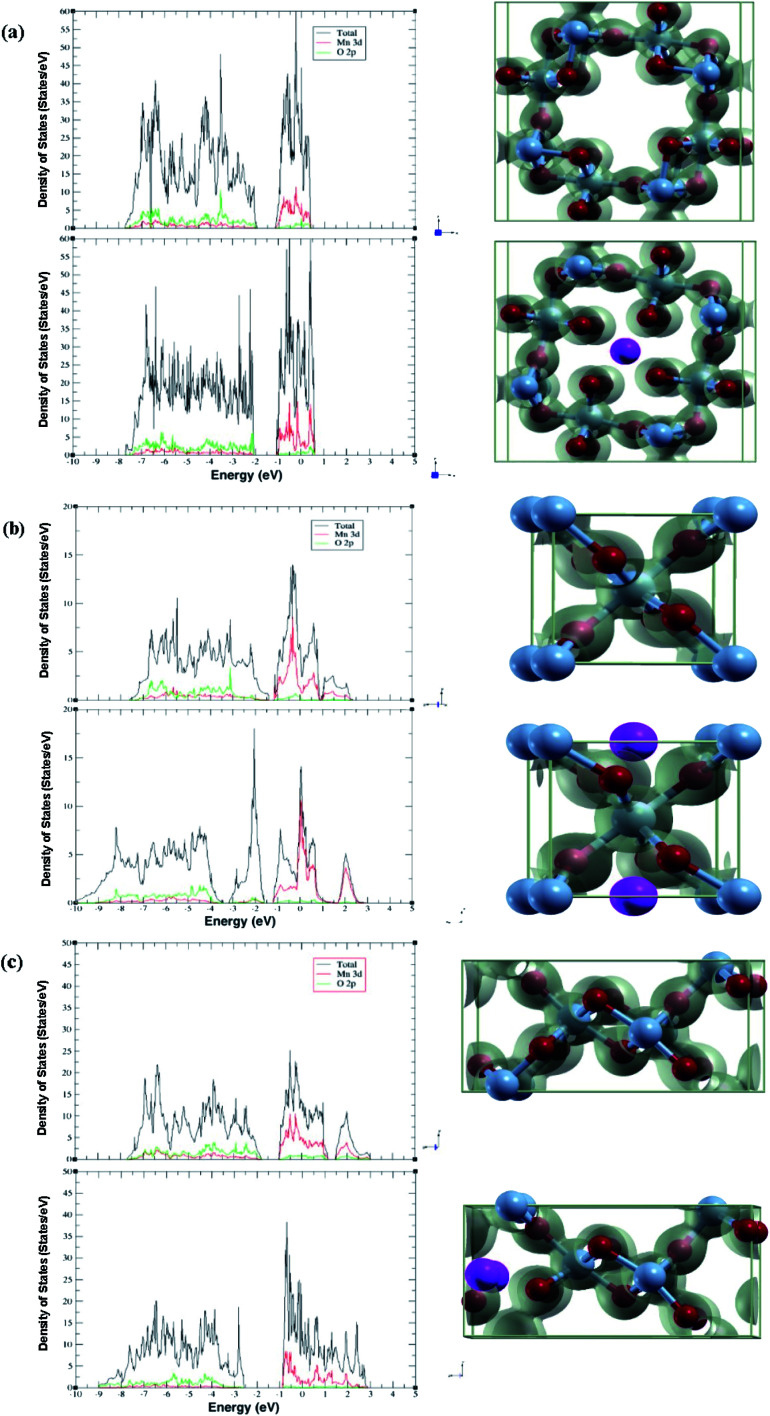
Calculated projected density of states and charge density comparing pre/post adsorption of Al-ion (top/bottom) in (a) α-MnO_2_, (b) β-MnO_2_, and (c) γ-MnO_2_. Charge density were calculated using a 0.08e isosurface.

The projected density of states gives insight into how each phase accommodates the Al atoms and shows charge localization. Due to not using U in our studies like other papers, the band gap calculated is an underestimation. Thus, in this section we will be looking at overall trends (Fig. 2).

In each phase, the conduction band is made of mostly Mn 3d states with a low number of O 2p states, while the valence band is more equal. Adding in an Al-ion adds an additional three electrons to fill up states. We can confirm this by comparing each polymorph before and after adsorption, where the conduction band decreases while the valence band increases in states. Our results show that more states are created in the conduction band. Likewise, the conduction band becomes more metallic with the exception of β-MnO_2_, which becomes more insulating.

This is supplemented by the charge density graph that can give a visualization on where the charge is localized. We see that for each phase after adsorption, more charge is localized around the nearest four oxygen atoms in addition to the four atoms moving closer to the Al.

### 3.3 Binding/diffusion energy

**Fig. 3:**
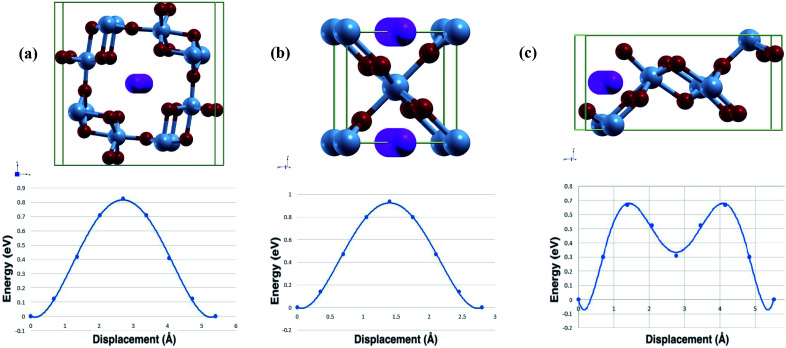
Calculated diffusion path and barrier (top/bottom) of Al-ion adsorption in (a) α-MnO_2_, (b) β-MnO_2_, and (c) γ-MnO_2_.

The diffusion path used in calculations and the calculated diffusion energy pathway can be seen in Fig. 3.

Both the binding energy and diffusion energy are important to gauge the speed of the kinetics of the reaction in the cathode of the battery. Slow kinetics may lead to lowered efficiency. The calculated binding energy and diffusion barrier were calculated and our results are shown in Table 3.

**Table 3:** Binding energy and diffusion barrier for Al-ion intercalated manganese dioxide

	α	β	γ
*B*_E_ (eV)	−0.666	−2.831	−2.930
*D*_B_ (eV)	0.810	0.923	0.672

As seen from the table, γ-MnO_2_ has both the lowest binding energy and diffusion barrier. α-MnO_2_ has the highest binding energy and the second lowest diffusion barrier. β-MnO_2_ accordingly has the highest diffusion barrier but moderate binding energy, although similar to γ-MnO_2_. The high diffusion energy may stem from the 1 × 1 tunnel that β has. The relatively smaller tunnel may result in worse kinetics. This is slightly offset by the volume change and the lower band gap leading to easier intercalation, hence the relatively low binding energy. All three phases have moderate diffusion barriers, while γ-MnO_2_ is close to that of Li-ion intercalation, which is the norm as of now.

## 4 Conclusion

In summary, we investigated each MnO_2_ polymorph with Al-ion intercalated and analyzed various properties. This included volume change, electronic structure, diffusion barrier, and binding energy. α-MnO_2_ goes through shrinkage after diffusion. Each polymorph except β-MnO_2_ becomes more metallic. γ-MnO_2_ has the lowest binding energy and diffusion barrier. Further research may investigate into specific polymorphs experimentally or computationally, or by changing the concentration of ions intercalated and exploring more deeply the magnetic effects or using other forms of calculations on Al-ion adsorption.

## Conflicts of interest

The authors declare no conflict of interest.

## Supplementary Material
